# From Psychosis to Coma: Diagnostic Pitfalls and Therapeutic Challenges in Anti-N-Methyl-D-Aspartate Receptor (Anti-NMDAR) Encephalitis Associated With Ovarian Teratoma

**DOI:** 10.7759/cureus.95676

**Published:** 2025-10-29

**Authors:** Thura Ko, Kaung Htet, Thet Myat Noe, May Thazin Htun, Moe Moe San, Thet Koko

**Affiliations:** 1 Specialty and Integrative Medicine, Leeds Teaching Hospitals NHS Trust, Leeds, GBR; 2 General Medicine, Leeds Teaching Hospitals NHS Trust, Leeds, GBR; 3 General Medicine, Liverpool University Hospitals NHS Foundation Trust, Liverpool, GBR; 4 Geriatrics, Poole Hospital, Poole, GBR; 5 Diabetes and Endocrinology, Airedale General Hospital, Steeton, GBR

**Keywords:** anti-nmdar encephalitis, immunotherapy, misdiagnosis, ovarian teratoma, teratoma resection, therapeutic dilemma

## Abstract

Anti-N-methyl-D-aspartate receptor (anti-NMDAR) encephalitis is a rare autoimmune disorder characterised by a spectrum of neuropsychiatric manifestations, movement and language disturbances, autonomic dysfunction, and coma. Its heterogeneous presentation poses a significant diagnostic challenge and is frequently misinterpreted as a primary psychiatric illness or infectious meningoencephalitis.

We present the case of a 26-year-old woman with an acute onset of prominent psychiatric symptoms, initially raising suspicion for a primary psychiatric disorder. The psychiatric team, however, suspected the possibility of an organic etiology, prompting empirical antiviral and antibiotic therapy for presumed infectious meningoencephalitis. Despite these treatments, her condition deteriorated, with declining Glasgow Coma Scale (GCS) scores, autonomic instability, and seizure-like activity, necessitating intensive care unit (ICU) admission. Immunotherapy was initiated for suspected autoimmune encephalitis, particularly in the context of negative cerebrospinal fluid (CSF) viral polymerase chain reaction (PCR) results. Further imaging revealed an ovarian teratoma. Due to limited response to first-line immunotherapy, rituximab was introduced, and the teratoma was surgically resected. This combined approach led to gradual but sustained clinical improvement. Later, the CSF autoimmune panel confirmed the presence of anti-NMDAR antibodies.

This case underscores the importance of considering anti-NMDAR encephalitis in patients presenting with acute psychiatric symptoms and highlights the value of a multidisciplinary approach in facilitating early diagnosis and treatment, thereby improving outcomes and reducing disability. It also discusses the therapeutic dilemma of initiating immunotherapy in the context of possible infectious meningoencephalitis and emphasises the dual role of teratoma removal in both acute management and relapse prevention.

## Introduction

Anti-N-methyl-D-aspartate receptor (anti-NMDAR) encephalitis is a B-cell-mediated autoimmune encephalitis in which the immunoglobulin G (IgG) antibodies, particularly IgG1 and IgG3, target against the NR1 subunit of the NMDAR in the central nervous system resulting in the internalisation of NMDAR, reduction of neuronal calcium influx, and decrease in the receptor-dependent synaptic currents [1,2].

The disease demonstrates a marked predilection for women, with a female-to-male ratio of approximately 4:1. The precise cause of anti-NMDAR encephalitis remains uncertain; however, it is associated with ovarian teratomas in approximately 60% of adult female patients and with viral infections, particularly in children following herpes simplex virus (HSV) encephalitis [3].

It is characterised by a constellation of neuropsychiatric symptoms, movement and language disturbances, autonomic dysfunction, and coma. Routine laboratory testing is usually non-specific. Head computed tomography (CT) is usually normal [4]. Consequently, it is often misdiagnosed as a primary psychiatric illness. Magnetic resonance imaging (MRI) of the brain is normal or demonstrates non-specific changes in approximately 70-80% of patients, with only a minority exhibiting features consistent with typical limbic encephalitis. However, CSF findings are abnormal in 80% with lymphocytic pleocytosis and unpaired oligoclonal bands. Anti-NMDAR antibodies are consistently detectable in CSF, where their presence is diagnostic of the disease, but are absent in approximately 20% of serum samples [3]. Electroencephalography (EEG) is abnormal in approximately 83.6% of patients, most commonly showing non-specific changes consistent with encephalopathy, with the rare occurrence of the distinctive extreme delta brush pattern warranting increased consideration of the diagnosis [5,6].

Given its substantial morbidity and mortality, the potential for prolonged hospitalisation, and the high rate of relapse, early initiation of aggressive therapy is advocated in patients with NMDAR antibody encephalitis. Immunotherapy, such as corticosteroids and intravenous immunoglobulin (IVIG), in conjunction with the prompt resection of associated teratomas when present, is considered essential to optimal management [3].

We present the case of a 26-year-old woman with anti-NMDAR encephalitis associated with an ovarian teratoma, illustrating the diagnostic and therapeutic challenges inherent to this condition. This report highlights the importance of maintaining a high index of clinical suspicion when initial manifestations resemble primary psychiatric illness and routine investigations are inconclusive, as well as the need for timely immunotherapy and prompt identification and removal of the underlying tumour to optimise patient outcomes.

This case was previously presented in poster format at the Society for Acute Medicine (SAM) Conference, Cambridge, May 8-9, 2025. 

## Case presentation

A 26-year-old woman with no significant past medical history presented to the Same Day Emergency Care (SDEC) unit with a one-week history of frontal headache, fever, and flu-like symptoms. Routine investigations, including a full blood count, liver and renal function tests, C-reactive protein (CRP), blood and urine culture, viral polymerase chain reaction (PCR) assays, and CT of the head, were unremarkable. In the absence of meningeal signs, including neck stiffness, photophobia, Brudzinski's sign, and Kernig's sign, the patient was discharged with a presumptive diagnosis of viral infection.

Three days later, she re-presented with acute behavioural changes, including nihilistic delusions, paranoid ideation, agitation, and thoughts of self-harm. She had no prior psychiatric history and denied substance or alcohol misuse. On examination, her Glasgow Coma Scale (GCS) score was 14/15 due to confusion. No focal neurological deficits or meningeal signs were identified. Repeat routine laboratory investigations were unremarkable. Given the absence of significant neurological findings and normal laboratory results, a primary psychiatric disorder was initially considered. However, urgent psychiatric review deemed the presentation inconsistent with a primary psychiatric illness, prompting further investigations to exclude infectious and autoimmune meningoencephalitis.

A lumbar puncture was attempted but unsuccessful on two occasions due to patient agitation; an MRI of the head was also not tolerated. Empirical acyclovir was therefore commenced for presumed viral meningoencephalitis, with plans to perform a lumbar puncture under anaesthesia. Two days later, lumbar puncture revealed lymphocytosis and moderately elevated protein levels, with a negative PCR result (Table 1). On microbiology and neurology advice, cefotaxime and amoxicillin were added to cover rarer central nervous system (CNS) infections, such as *Listeria* and *Mycoplasma*, due to an unsatisfactory response to acyclovir. Concurrently, cerebrospinal fluid (CSF) and serum infection screens (Table 2), along with autoimmune panels (Table 3), were sent. An ultrasound of the abdomen and pelvis was arranged to evaluate for ovarian pathology, given its recognised association with autoimmune encephalitis in young women.

**Table 1:** CSF analysis WBC: white blood cell; RBC: red blood cell; HSV: herpes simplex virus; VZV: varicella zoster virus; PCR: polymerase chain reaction; CSF: cerebrospinal fluid

Parameters	Result	Reference value
Appearance	Clear	Clear
Colour	Colourless fluid	Colourless
Gram stain	No organisms	-
WBC	104 cells×10^6^/L	0-5 cells×10^6^/L
RBC	<1 cells×10^6^/L	0-5 cells×10^6^/L
Neutrophils	1 cell×10^6^/L	0-5 cells×10^6^/L
Lymphocytes	99 cells×10^6^/L	0-5 cells×10^6^/L
Culture	No growth	-
CSF glucose	2.7 mmol/L	2.2-3.9 mmol/L
CSF protein	0.86 g/L	0.20-0.40 g/L
Adenovirus PCR	Not detected	-
HSV PCR	Not detected	-
VZV PCR	Not detected	-
*Enterovirus* PCR	Not detected	-
*Parechovirus* PCR	Not detected	-
*Neisseria meningitidis* PCR	Not detected	-
*Streptococcus pneumoniae* PCR	Not detected	-

**Table 2:** Serology test result VZV IgG Ab: varicella zoster virus immunoglobulin G antibodies; HIV: human immunodeficiency virus

Test	Result	Reference value
Cytomegalovirus	Not detected	Negative
Epstein-Barr virus	Not detected	Negative
Hepatitis B, C, and E	Not detected	Negative
Syphilis antibody	Not detected	Negative
Leptospira serology	Not detected	Negative
VZV IgG Ab	Positive (≥150 mIU/mL)	≥150 mIU/mL (evidence of immunity to VZV from past infection or immunisation)
Adenovirus	Not detected	Negative
Borrelia	Not detected	Negative
Toxoplasma	Not detected	Negative
Herpes simplex virus	Not detected	Negative
HIV	Not detected	Negative

**Table 3:** Antibody test result (serum) Anti-CV2/CRMP5 Ab: anti-CV2/collapsin response mediator protein 5 antibody; AMPA: α-amino-3-hydroxy-5-methyl-4-isoxazolepropionic acid; GABAb: gamma-aminobutyric acid type b receptor; LGI1: leucine-rich glioma-inactivated 1; CASPR2: contactin-associated protein-like 2; ANCA: anti-neutrophil cytoplasmic antibody; ANA: antinuclear antibody; VGCC: voltage-gated calcium channel

Component	Result	Reference value
Anti-Hu Ab	Negative	Negative
Anti-Yo Ab	Negative	Negative
Anti-Ri Ab	Negative	Negative
Anti-Ma2/Ta Ab	Negative	Negative
Anti-CV2/CRMP5 Ab	Negative	Negative
Anti-amphiphysin Ab	Negative	Negative
AMPA1 Ab	Negative	Negative
AMPA2 Ab	Negative	Negative
GABAb	Negative	Negative
LGI1 Ab	Negative	Negative
CASPR2 Ab	Negative	Negative
ANCA	Negative	Negative
ANA	Negative	Negative
Streptolysin O Ab	227 IU/mL	0-408 IU/mL
IgG	15.8 g/L	6.0-16.0 g/L
IgA	1.34 g/L	0.80-2.80 g/L
IgM	0.56 g/L	0.50-1.90 g/L
Complement C3	1.29 g/L	0.75-1.65 g/L
Complement C4	0.18 g/L	0.14-0.54 g/L
VGCC Ab	<45 pM	0-45 pM

On day 5 of admission, the patient experienced three seizure-like episodes characterised by stiffening of the arms, head retraction, and an arching posture. A repeat CT of the head was unremarkable. EEG showed non-specific diffuse cortical dysfunction, without evidence of non-convulsive status epilepticus or subclinical seizures. Levetiracetam was initiated at a dose of 500 mg every 12 hours and gradually titrated to 2000 mg twice daily. Subsequently, the patient deteriorated further with worsening GCS, autonomic instability, and recurrent seizure-like episodes, necessitating intensive care unit (ICU) admission.

**Figure 1:**
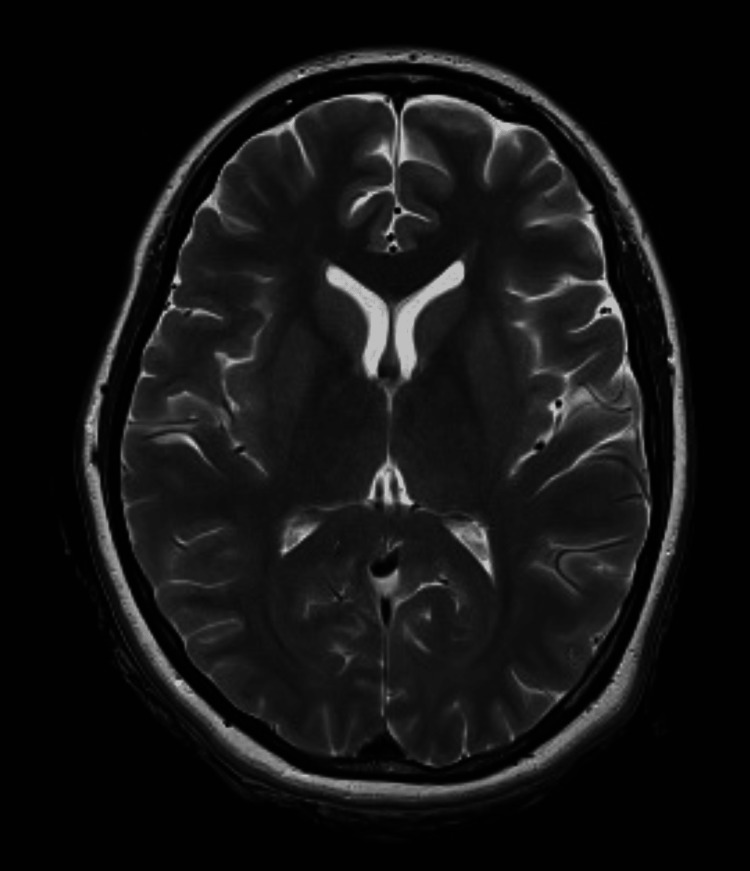
MRI of the brain (axial T2-weighted image) showing normal findings Normal intracranial appearances for age, with no parenchymal signal abnormality or structural lesion MRI: magnetic resonance imaging

**Figure 2:**
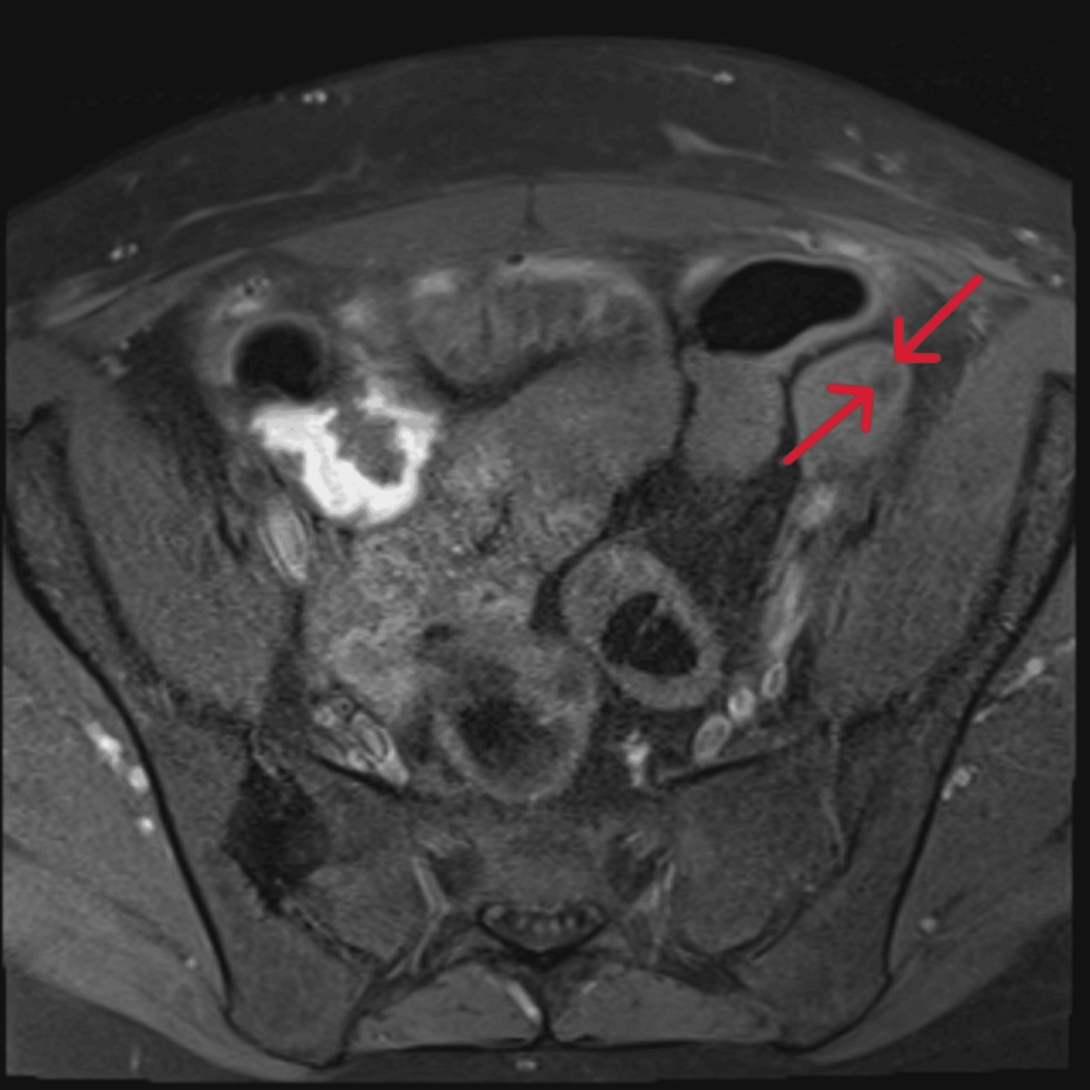
MRI of the pelvis demonstrating left ovarian teratoma Coronal T2-weighted MRI of the pelvis highlights a heterogeneous left adnexal mass (red arrows), consistent with a left ovarian teratoma MRI: magnetic resonance imaging

During her ICU stay, an MRI of the brain was performed and was normal (Figure 1). However, abdominal ultrasonography demonstrated an 11 mm echogenic lesion within the left ovary, possibly representing a small intra-ovarian dermoid/teratoma. Notably, a pelvic ultrasound performed six months earlier had shown normal ovaries. Suspicion for autoimmune encephalitis was therefore heightened. High-dose intravenous methylprednisolone (1 g daily) was initiated on day 6, followed by IVIG at a dose of 0.4 mg/kg/day for five consecutive days, commencing on day 9, in view of ongoing clinical deterioration despite concurrent antiviral and antibiotic therapy. Additional antiseizure agents were also introduced. These included a continuous midazolam infusion, initiated at 0.05-0.2 mg/kg/hour and titrated according to clinical response; phenytoin, administered as a loading dose of 15-20 mg/kg followed by a maintenance dose of 300 mg daily in divided doses; and lacosamide, given as a loading dose of 200 mg followed by 100 mg twice daily and titrated as required. CT of the thorax, abdomen, and pelvis and MRI of the pelvis (Figure 2) were requested to further characterise the ovarian lesion, and gynaecology input was sought.

As there was no clinical improvement with first-line immunotherapy, rituximab was initiated on day 17 at a dose of 1,000 mg intravenously every two weeks for two doses. While awaiting CSF antibody results, the clinical suspicion for anti-NMDAR encephalitis was considered high, and laparoscopic oophorectomy was performed on day 19 following MRI confirmation of the ovarian lesion/teratoma. Around this time, serum testing returned positive for NMDAR antibodies. Five days later, CSF analysis confirmed the presence of NMDAR antibodies, establishing the diagnosis (Table 4).

**Table 4:** Diagnostic anti-NMDAR antibody result NMDAR: N-methyl-D-aspartate receptor; LGI1: leucine-rich glioma-inactivated 1; CASPR2: contactin-associated protein-like 2

Test	Result	Reference value
LGI1 Ab CSF	Negative	Negative
CASPR2 Ab CSF	Negative	Negative
Fixed NMDAR Ab CSF	Positive	Negative
Fixed NMDAR Ab serum	Positive	Negative

Following the initiation of second-line immunotherapy and tumour removal, the patient demonstrated gradual clinical improvement. After a prolonged admission requiring three months of hospitalisation and intensive rehabilitation, she was discharged with significant recovery. At the time of discharge, the patient demonstrated substantial neurological recovery. She was independently mobile, with a modified Rankin Scale (mRS) score of 1, and had regained full independence in activities of daily living. Mild short-term memory difficulty persisted, characterised by occasional lapses in recalling recent conversations or events from preceding days. During cognitive rehabilitation, she demonstrated effective use of external memory aids (e.g., written notes) to recall therapy content independently. Mild confusion was occasionally observed when differentiating between multiple sessions, consistent with residual post-encephalitic memory impairment; however, her cognitive function continued to improve over subsequent follow-up visits.

At the most recent follow-up, approximately two months after discharge, she remained neurologically stable and functionally independent, with no evidence of relapse or new neurological deficits. Her discharge medications included cholecalciferol 800 IU once daily, alendronic acid 70 mg once weekly, lansoprazole 30 mg once daily, and a tapering regimen of prednisolone 20 mg once daily alongside levetiracetam 1 g daily. At seven months following hospital discharge, she has successfully tapered prednisolone to 5 mg daily and has been completely weaned off levetiracetam without subsequent clinical relapses. 

## Discussion

Anti-NMDAR encephalitis poses significant diagnostic challenges due to its heterogeneous clinical presentation and the non-specific nature of early investigative findings, which may contribute to the delayed initiation of immunotherapy and poorer outcomes. In the initial stage, approximately 40-70% of patients present with non-specific symptoms such as headache and fever, followed within days to weeks by psychiatric manifestations in up to 90% of cases, often mimicking primary psychiatric disorders [7,8]. As the disease progresses, rapid neurological deterioration typically ensues, including movement and language disturbances, autonomic dysfunction, seizures, and, in severe cases, coma [3,7,9-11]. Moreover, initial diagnostic investigations are often inconclusive, with MRI of the brain and EEG demonstrating non-specific findings in nearly 80% of cases [3,5,6]. Although CSF analysis may reveal lymphocytic pleocytosis, this finding lacks specificity for anti-NMDAR encephalitis. Consequently, even when autoimmune encephalitis is suspected at an early stage, initiation of immunotherapy may be delayed by the need to exclude alternative diagnoses, particularly infectious meningoencephalitis, and by the time required to obtain confirmatory antibody results.

This was evident in our case, where a viral illness was initially suspected at the first presentation. Although one might argue that lumbar puncture and CSF sampling were not performed, this decision was reasonable given the absence of significant neurological symptoms, unremarkable routine laboratory tests, and a normal head CT. Even if CSF had been obtained, the likely finding of lymphocytosis could have led to a misdiagnosis of viral meningitis, a typically self-limiting condition. During the second presentation, primary psychiatric disease was initially considered, further reflecting the diagnostic complexity.

The management of anti-NMDAR encephalitis is equally as complex as its diagnosis and necessitates a multidisciplinary team approach. Expeditious initiation of immunotherapy can improve outcomes and reduce relapses [7,9,12]. Favourable outcomes (mRS ≤2) are more likely with prompt treatment, whereas delays beyond four weeks are associated with poorer functional outcomes at one year [3,9,13]. Although therapy may be deferred pending infectious exclusion, empirical treatment may be commenced before the definitive diagnosis when there is a reasonable degree of suspicion [14]. For this reason, the timely involvement of a neurologist is essential to facilitate rapid diagnosis and treatment initiation. Psychiatric input is valuable in differentiating primary psychiatric disorders from organic disease. Intensive care support may be required during severe stages of illness for airway protection, stabilisation of autonomic dysfunction, seizure management, and reduced consciousness [15]. A thorough investigation for an underlying teratoma is critical due to its well-established association with anti-NMDAR encephalitis, with such tumours identified in approximately 30-60% of female patients of reproductive age, as reported by Banach et al. [16]. However, the reported prevalence varies across studies, ranging from 36% to 50% in another cohort [17], likely reflecting differences in patient age groups, geographic regions, and study methodologies. If detected, consultation with obstetrics and gynaecology should be sought. Surgical resection of the teratoma is considered a cornerstone of therapy, with efficacy comparable to first-line immunotherapy [3,18], and is important not only for acute disease control but also for relapse prevention [3,9]. Approximately 50% of patients demonstrate clinical improvement following first-line immunotherapy with corticosteroids and IVIG. An additional 20% exhibit improvement after receiving second-line immunotherapy. Overall, with appropriate immunotherapy and timely tumour resection, approximately 80% of patients achieve an mRS score of 0-2 within 24 months [3].

In the present case, proactive involvement of psychiatry, neurology, and other specialties facilitated swift recognition and management. First-line immunotherapy with corticosteroids and IVIG was administered on days 6 and 9 of admission, respectively, followed by second-line immunotherapy and teratoma resection. These interventions collectively contributed to the patient's gradual clinical improvement and recovery.

## Conclusions

This case highlights the diagnostic and therapeutic complexity of anti-NMDAR encephalitis and emphasises the importance of considering this condition in patients presenting with acute psychiatric symptoms. It underscores the value of cross-specialty collaboration in achieving prompt diagnosis and effective management, the critical role of early immunotherapy in improving outcomes and reducing disability, the justification for empirical treatment when clinical suspicion is high and infectious causes are reasonably excluded, and the necessity of tumour resection when an associated teratoma is identified.

## References

[1] Dalmau J, Gleichman AJ, Hughes EG (2008). Anti-NMDA-receptor encephalitis: case series and analysis of the effects of antibodies. Lancet Neurol.

[2] Hughes EG, Peng X, Gleichman AJ (2010). Cellular and synaptic mechanisms of anti-NMDA receptor encephalitis. J Neurosci.

[3] Uy CE, Binks S, Irani SR (2021). Autoimmune encephalitis: clinical spectrum and management. Pract Neurol.

[4] Rosenfeld MR, Dalmau J (2011). Anti-NMDA-receptor encephalitis and other synaptic autoimmune disorders. Curr Treat Options Neurol.

[5] Gillinder L, Warren N, Hartel G, Dionisio S, O'Gorman C (2019). EEG findings in NMDA encephalitis - a systematic review. Seizure.

[6] Schmitt SE, Pargeon K, Frechette ES, Hirsch LJ, Dalmau J, Friedman D (2012). Extreme delta brush: a unique EEG pattern in adults with anti-NMDA receptor encephalitis. Neurology.

[7] Nguyen L, Wang C (2023). Anti-NMDA receptor autoimmune encephalitis: diagnosis and management strategies. Int J Gen Med.

[8] Dalmau J, Armangué T, Planagumà J (2019). An update on anti-NMDA receptor encephalitis for neurologists and psychiatrists: mechanisms and models. Lancet Neurol.

[9] Titulaer MJ, McCracken L, Gabilondo I (2013). Treatment and prognostic factors for long-term outcome in patients with anti-NMDA receptor encephalitis: an observational cohort study. Lancet Neurol.

[10] Xu X, Lu Q, Huang Y (2020). Anti-NMDAR encephalitis: a single-center, longitudinal study in China. Neurol Neuroimmunol Neuroinflamm.

[11] Gong X, Chen C, Liu X (2021). Long-term functional outcomes and relapse of anti-NMDA receptor encephalitis: a cohort study in Western China. Neurol Neuroimmunol Neuroinflamm.

[12] Nosadini M, Eyre M, Molteni E (2021). Use and safety of immunotherapeutic management of N-methyl-d-aspartate receptor antibody encephalitis: a meta-analysis. JAMA Neurol.

[13] Balu R, McCracken L, Lancaster E, Graus F, Dalmau J, Titulaer MJ (2019). A score that predicts 1-year functional status in patients with anti-NMDA receptor encephalitis. Neurology.

[14] Breese EH, Dalmau J, Lennon VA, Apiwattanakul M, Sokol DK (2010). Anti-N-methyl-D-aspartate receptor encephalitis: early treatment is beneficial. Pediatr Neurol.

[15] Neyens RR, Gaskill GE, Chalela JA (2018). Critical care management of anti-N-methyl-D-aspartate receptor encephalitis. Crit Care Med.

[16] Banach W, Banach P, Szweda H (2024). Ovarian teratoma-associated anti-NMDAR encephalitis in women with first-time neuropsychiatric symptoms: a meta-analysis and systematic review of reported cases. Heliyon.

[17] Martin AL, Jolliffe E, Hertweck SP (2018). Ovarian teratoma associated with coexisting anti-N-methyl-D-aspartate receptor and glial fibrillary acidic protein autoimmune meningoencephalitis in an adolescent girl: a case report. J Pediatr Adolesc Gynecol.

[18] Makuch M, Wilson R, Al-Diwani A (2018). N-methyl-D-aspartate receptor antibody production from germinal center reactions: therapeutic implications. Ann Neurol.

